# Comparative Analysis of Gut Microbiota Among Captive Waterbird Species: Effects of Diet and Environmental Factors

**DOI:** 10.1002/vms3.70865

**Published:** 2026-04-13

**Authors:** He Liu, Tingbei Bo, Jing Li, Yuekun Zhang, Huanzhi Zhou

**Affiliations:** ^1^ Beijing Key Laboratory of Captive Wildlife Technology Beijing Zoo Beijing China; ^2^ School of Grassland Science Beijing Forestry University Beijing China

**Keywords:** artificial lake, captivity, gut microbiota, waterbirds

## Abstract

The gut microbiota of avian species is influenced by a diverse array of factors, encompassing host genetics, environmental conditions, physiological states, dietary patterns and age, among others. With the advent of high‐throughput sequencing technology, research into avian gut microbiota has gained momentum. The artificial lake within the zoo serves as a unique nexus between the natural and captive environments. Despite this, our understanding of the gut microbiota of waterbirds residing in such an artificial habitats remains limited. In this study, we focused on three distinct species of waterbird kept in the artificial lake and traditional enclosures. We used high‐throughput sequencing technology to determine the faecal microbiome of 25 waterbirds from three species, including bar‐headed goose (*Anser indicus*), ruddy shelduck (*Tadorna ferruginea*) and black‐necked crane (*Grus nigricollis*). Our objective was to elucidate the composition and community structure of their gut microbiota, while exploring the nexus between dietary habits and gut microbiota. Our findings revealed that the dietary patterns and gut microbiota of ruddy shelducks and bar‐headed geese exhibited similar traits. On the other hand, black‐necked cranes, whose food primarily consists of fish and meat, possessed a gut microbiota optimized for the digestion of protein and fat. Notably, artificial lakes may support higher microbial diversity compared to cage feeding. The results underscore the significance of diet as the primary determinant of gut microbiota variation among the captive waterbird. This study provides implications for promoting the healthy growth and well‐being of wild waterbird in captivity.

## Graphical abstract

This study reveals that diet drives gut microbiota differences in captive waterbirds (bar‐headed goose, ruddy shelduck, black‐necked crane), with protein‐rich diets shaping distinct microbial communities. Artificial lakes enhance microbial diversity compared to enclosures, offering insights for improving captive waterbird health.

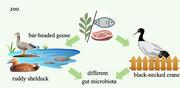

## 1 Introduction

Gut microbiota plays a crucial role in the health of animal hosts (Kohl 2012; Waite and Taylor 2014). Many factors like genetics, diet, immune status and environment can influence gut microbial diversity (Faith et al. 2011). Diet plays a very important role in the formation of gut microbiota, and dietary changes can cause significant changes in gut microbiota within 24 h (Singh et al. 2017). Small number of diet‐manipulation studies have demonstrated that avian microbiomes respond to nutritionally and compositionally different diets. Feeding different diets can cause changes in the gut microbiota of wild‐caught great tits (*Parus major*) and reshape individual behaviour (Davidson et al. 2020; Bodawatta et al. 2021). Diet is an important factor leading to differences in the gut microbiota of tree sparrows (*Passer montanus*) along the urbanization gradient (Teyssier et al. 2020). In addition, gut bacteria can also facilitate detoxification of food items, allowing bird hosts to feed on otherwise toxic diets (Kohl et al. 2016).

With the development of high‐throughput sequencing technology, we have made great progress in the study of wildlife gut microbiota. In recent years, there have been many studies on avian gut microbiota. Birds are a group of strong adaptability to environment, with complex life history, diversified feeding habits and migration (Kohl 2012). Numerous studies have examined how factors such as variations between bird species, age, captivity, and viral infections influence the gut microbiota (Barbosa et al. 2016; Wang et al. 2016; Zhao et al. 2018; Hubalek 2004). In the study of waterfowl on the Qinghai Tibet Plateau (Bo et al. 2022), we found that altitude is the main factor that affects the proportion of key bacteria. With an increase in altitude, the proportion of Firmicutes, including *Lactobacillus* and *Lachnospiraceae*, in brown‐headed gulls decreases (Bo et al. 2022).

Recent studies indicate that there are marked discrepancies in the gut microbiota of wild versus captive mammals, attributable largely to alterations in living conditions and dietary regimes under captivity (Gibson et al. 2019; Guo et al. 2019; Ning et al. 2020). For instance, in captivity, the limited space for animals to live, the single source of diet, and the lack of communication between individuals can have a negative impact on the composition of their gut microbiota (McKenzie et al. 2017; Frankel et al. 2019). For example, captive environment reduces microbiota richness and diversity compared to wild individuals in red colobus monkey (*Procolobus gordonorum*) and Andean bear (*Tremarctos ornatus*) (Barelli et al. 2015; Andrea et al. 2017). In fact, the impact of cage breeding on birds may be more serious, because most birds require more space to fly. The gut microbiota composition of captive and wild oriental white stork (*Ciconia boyciana*) was similar, but the abundances were significantly different, and the gut microbiota was related to the host's metabolic pathways (Wu et al. 2021). In response to these findings, an increasing number of urban parks and zoos are incorporating waterfowl lakes to provide an open environment beyond the constraints of cages. Contrary to conventional poultry breeding in enclosures, these lakes offer a semi‐natural habitat for waterbirds. However, the scrutiny of gut microbiota within these semi‐natural captive settings has been relatively overlooked, warranting further investigation.

The black‐necked crane, a member of the Gruidae family, inhabits the high‐altitude wetlands of the Qinghai–Tibet and Yunnan–Guizhou plateaus in China (H. T. Song et al. 2014). This species follows a distinct breeding pattern, reproducing on the elevated Qinghai–Tibet Plateau during the summer months and migrating to the lower‐altitude middle reaches of the Yarlung Zangbo River during winter. The black‐necked crane is categorized as “vulnerable” on the IUCN Red List of Threatened Species due to various anthropogenic pressures (IUCN 2018). In contrast, the ruddy shelduck and the bar‐headed goose, both belonging to the *Anatidae* family, exhibit a wide distribution range and migrate seasonally. Their dietary preferences encompass aquatic plant leaves, buds, seeds, as well as insects, shrimps and small fish. Notably, these two species are listed as ‘least concern’ on the IUCN Red List of Threatened Species, indicating a relatively stable population status (IUCN 2018). In the present study, we elucidated the composition and community structure of the three bird species gut microbiota, while exploring the nexus between dietary habits and gut microbiota. We found that the dietary patterns and gut microbiota exhibited significant correlation. In addition, artificial lakes may support higher microbial diversity compared to cage feeding. Our results underscore the significance of diet as the primary determinant of gut microbiota variation among the captive waterbird. This study provides implications for promoting the healthy growth and well‐being of wild waterbirds in captivity.

## 2 Methods

### 2.1 Animals and Sample Collection

Faecal samples of bar‐headed geese (*n* = 6), ruddy shelducks (*n* = 6) and black‐necked cranes (*n* = 13) were collected in Beijing zoo during September 2021. The bar‐headed geese and ruddy shelducks live in waterfowl lakes, while the black‐necked cranes lives in captive house (a kind of display cage of the zoo, Figure S1). We obtained the daily energy intake and food composition of different birds from the veterinarian at the zoo, which was shown in Table S1.

Birds were driven into a cage with sterile plastic film at the bottom, without caught or bound. After adapting for 3–4 h, they were very quiet and stress free. Then the fresh faeces were picked up from the film. The faeces were put into the 2 mL sterilized storage tubes and stored in liquid nitrogen immediately. Finally, samples were transported in dried ice for transportation and stored in −80°C lab freezer. This study was approved by the faculty of the Beijing Zoo and Institute of Zoology, Chinese Academy of Sciences (IOZ‐IACUC‐2022‐114).

### 2.2 16S rRNA Gene Sequencing Analysis

DNA from faecal contents was extracted by QIAamp DNA stool Mini Kit from Qiagen (Hilden, Germany). The 16S rRNA gene comprising V3 and V4 regions was amplified by PCR using composite specific bacterial primers (338F 5′‐ACTCCTACGGGAGGCAGCA‐3′; 806R 5′‐GGACTACHVGGGTWTCTAAT‐3). All PCR reactions were carried out with 15 µL of Phusion High‐Fidelity PCR Master Mix (New England Biolabs), 2 µM of each primer and approximately 10 ng of template DNA. Thermal cycling conditions were as follows: Initial denaturation at 98°C for 1 min; 30 cycles of denaturation at 98°C for 10 s, annealing at 50°C for 30 s and extension at 72°C for 30 s; followed by a final extension at 72°C for 5 min. PCR products were visualized by electrophoresis on a 2% agarose gel in 1× TAE buffer. Amplified products were then pooled in equimolar concentrations and purified using the Universal DNA Purification Kit (TianGen, China). High‐throughput pyrosequencing of the PCR products was performed on an Illumina NovaSeq 6000 platform at Biomarker Technologies Co., Ltd. (Shenzhen, China). Trimmomatic (v0.33) (Bolger et al. 2014) was used to filter the Raw Reads obtained by sequencing, Cutadapt 1.9.1 software (Quast et al. 2013) was used to identify and remove primer sequences and UCHIME v4.2 software (Edgar et al. 2011) was used to remove chimeric sequences. Usearch software (11.0.667) (Edgar 2010) was used to cluster Reads at 97.0% similarity level to obtain operational taxonomic units (OTUs).

For alpha diversity analysis, we rarified the OTU to several metrics, including Chao1 and Shannon index. For beta diversity analysis principal coordinate analysis (PCoA), was used with bray_curtis and weighted/unweighted UniFrac distance. The LDA effect size (LEfSe) analysis was performed for the quantitative analysis of biomarkers among each group (LDA threshold of >2). LEfSe analysis was used to identify significantly enriched genus in each group. A calculated *p*‐value < 0.05 was considered to be statistically significant. PICRUSt was used to predict the functions of microbial communities (Langille et al. 2013). Predicted functional pathways were annotated using the Kyoto Encyclopedia of Genes and Genomes (KEGG) database (Kanehisa et al. 2012).

### 2.3 Statistical Analysis

Alpha diversity and abundance were presented as means ± SEM. The beta diversity statistical analysis was performed using ANOSIM. The correlation between microbiome and diet components was analysed by Spearman correlation. Differences between groups were statistically analysed using Kruskal–Wallis test with the significance level of *p* < 0.05. (*p < 0.05, **p < 0.01, ***p < 0.001).

## 3 Results

**FIGURE 1:**
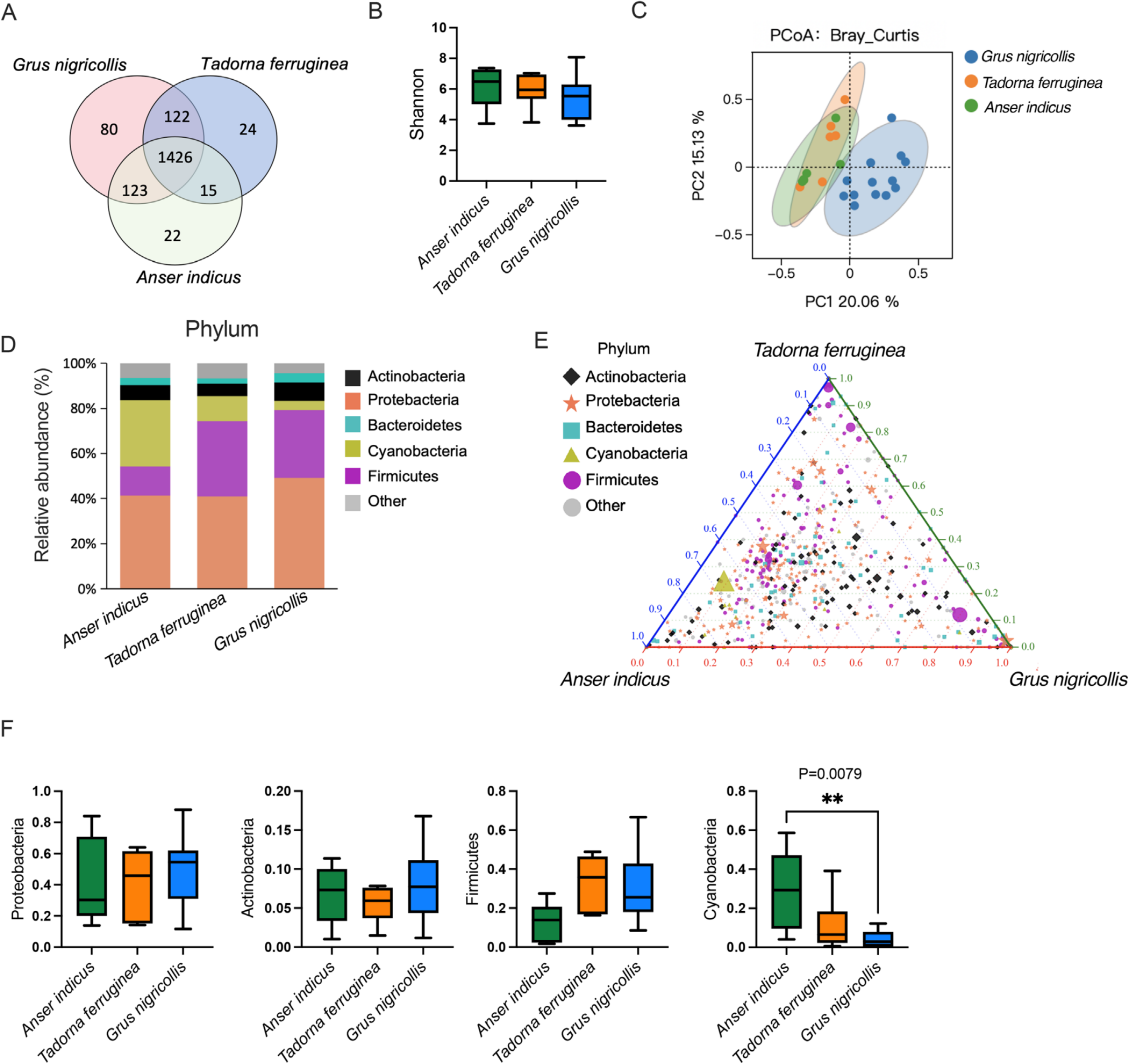
Results of gut microbiota of *Grus nigricollis*, *Tadorna ferruginea* and *Anser indicus*. (**A**) Venn diagram showing OTUs of the 3 species. (**B**) Alpha diversity (Shannon index) of gut microbiota. (**C**) Principal coordinates (PCoA) analysis with Bray–Curtis distance calculated using relative abundance at OTUs levels among three species. Each ellipse in the PCoA was plotted with a 95% confidence interval. (**D**) Taxonomy composition of gut microbiota at the phylum level of three bird species. (**E**) Ternary plots showing the relative abundance of all gut microbial phyla for the three bird species. Each point corresponds to a phylum. (**F**) The relative abundance of four phyla across different waterbirds. Data are means ± SEM. **p < 0.01.

Illumina NovaSeq sequencing of the V3–V4 regions of the bacterial 16S rDNA yielded a total of 1,991,995 pairs raw reads. A total of 1,985,503 clean reads were obtained after trimming adapter sequences and filtering out low‐quality reads, and possibly chimeric sequences and host DNA. Each sample produces at least 72,841 clean reads, with an average of 79,420 clean reads (Table S2). All the quality filtered sequences clustered in 1812 OTUs with a minimum abundance of 0.01%. On average, there were 907 OTUs for each sample (Table S3). A Venn diagram showed that a total of 1426 OTUs were identified as core bacterial OTUs in the 3 species. The number of unique OTUs in each group was 80 (black‐necked crane), 22 (bar‐headed goose), 24 (ruddy shelduck), respectively (Figure 1A).

The alpha diversity measures (Chao 1 index and Shannon diversity index) were calculated in each group to examine whether the three species differed in alpha diversity. Statistical analyses showed no significant difference in alpha diversity among the three species (Figure 1B and Figure S2A). In PCoA analysis, gut microbial assemblages were well clustered in bar‐headed geese and ruddy shelducks, while the black‐necked cranes was far away from them (ANOSIM, bray_curtis, *R* = 0.472, *p* = 0.001, Figure 1C and Figure S2B,C). This indicated significant differences in the gut microbiota structure among the three bird species. The microbial structure clustering heatmap among the samples also reflects that the black‐necked cranes were different from bar‐headed geese and ruddy shelducks, and the microbiota structures of the latter two waterbirds were similar (Figure S2D). Microbial composition among three bird species almost overlapped at the phylum level, but their dominant bacteria are different. Cyanobacteria were the most common in bar‐headed geese, Firmicutes was abundant in ruddy shelducks, while Proteobacteria predominated in the gut of black‐necked cranes (Figure 1D,E). Statistical analysis also showed that the abundance of Cyanobacteria was significantly different between the bar‐headed geese and black‐necked cranes (*p* = 0.0079, Figure 1F).

**FIGURE 2:**
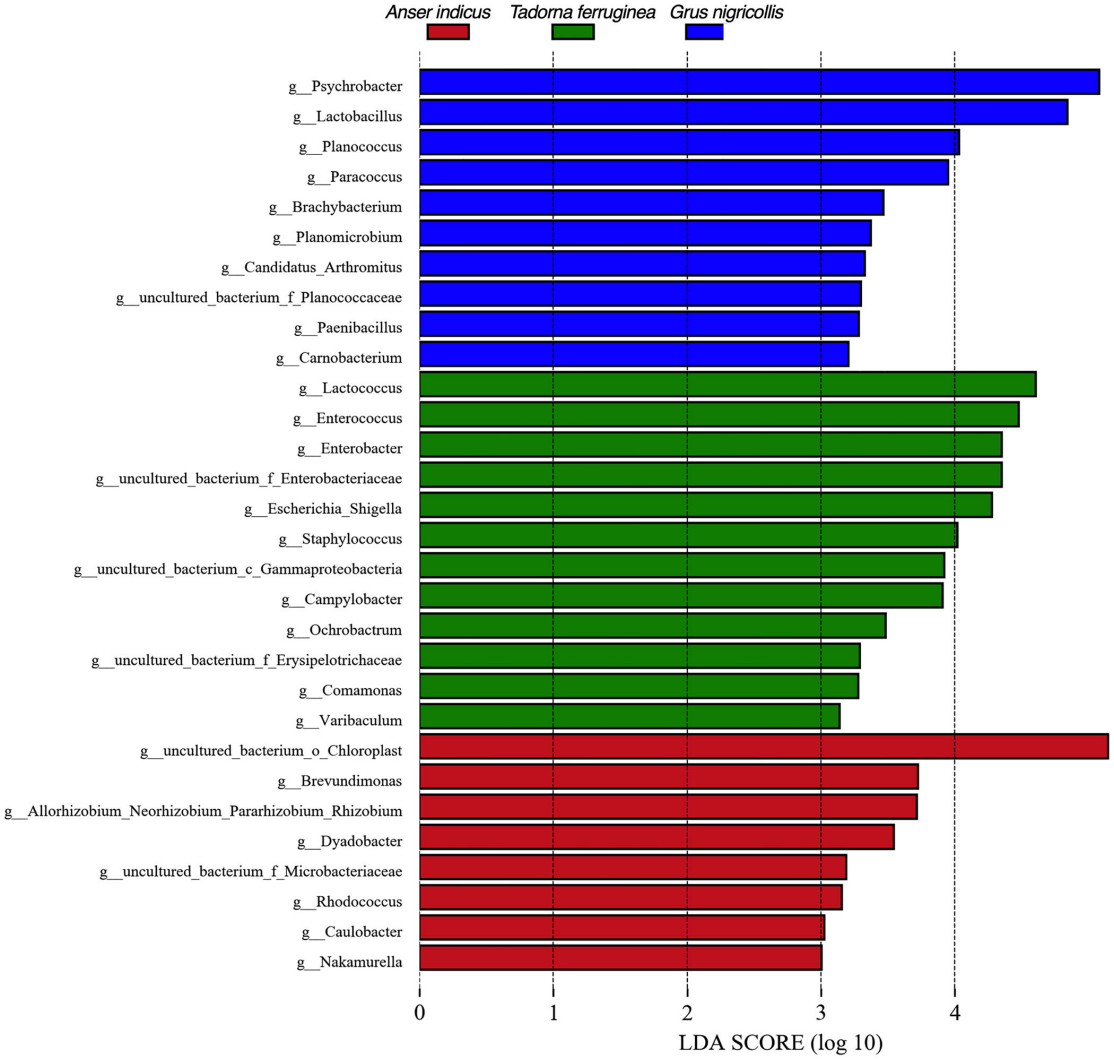
LDA scores of the differentially abundant taxa enriched in microbiota (taxa with LDA score > 3 and a significance of *p* < 0.05 are shown). (g_means genus level, f_means family level).

Among these core OTUs of three species, the top ten most abundant core genera were *Psychrobacter*, *Lactobacillus*, *Acinetobacter*, *Enterobacter*, *Lactococcus*, *Escherichia‐Shigella*, *Enterococcus*, *Sphingomonas* (Table S4). At the genus level, ruddy shelducks was mainly dominated by *Lactococcus*, *Enterococcus*, *Enterbacter*, *Escherichia_Shigella*, *Campylobacter*, *Ochrobactrum*, *Comamonas*, *Varibaculum* and *Staphylococcus*, while *Psychrobacter*, *Lactobacillus*, *Planococcus*, *Paracoccus*, *Brachybacterium*, *Planomicrobium*, *Paenibacillus* and *Carnobacterium* were abundant in black‐necked cranes. In bar‐headed geese, the *Brevundimonas*, *Dyadobacter*, *Rhodococcus*, *Caulobacter* and *Nakamurella* were abundant (LDA>3, Figure 2).

**FIGURE 3:**
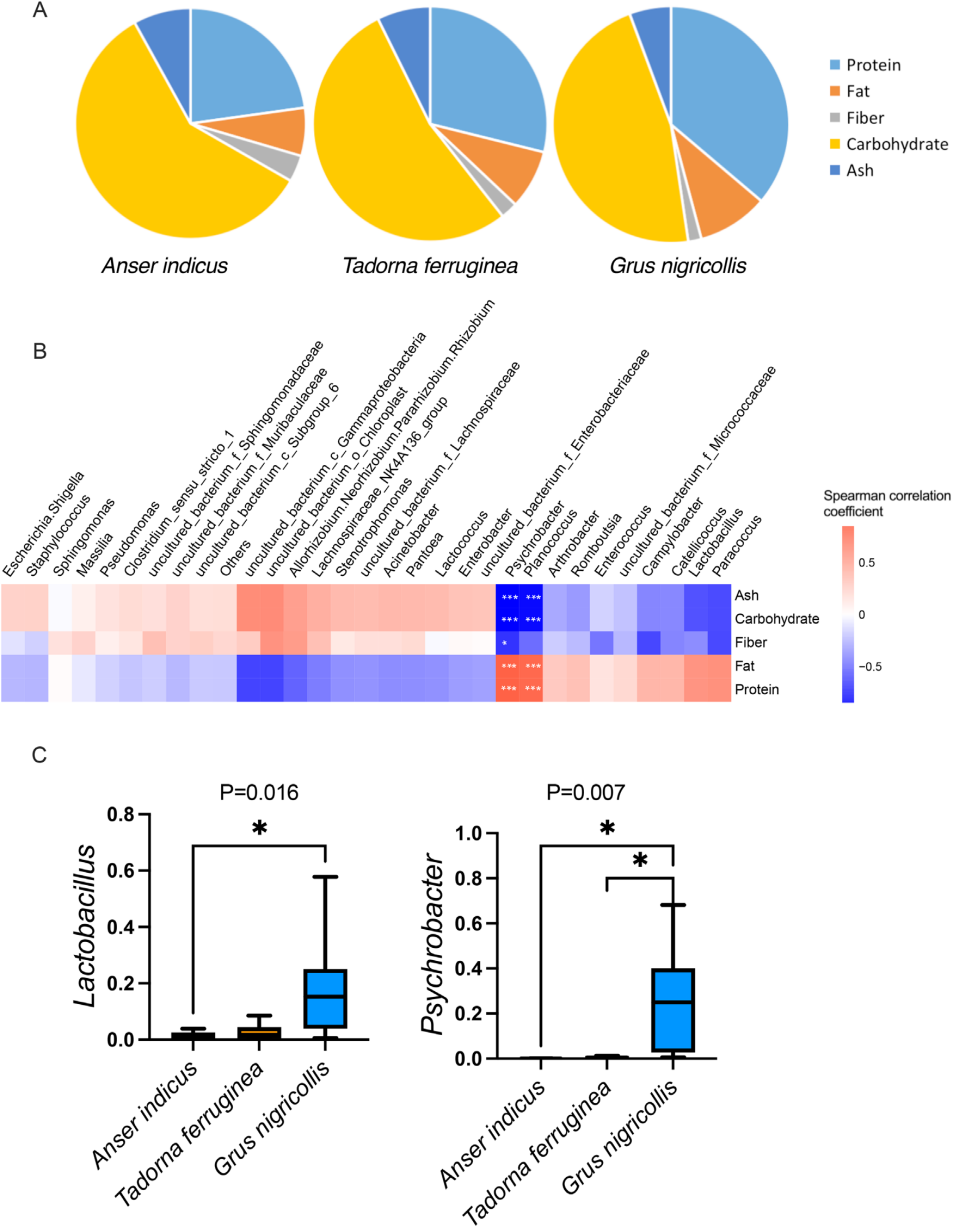
Relationship between feeding habits of birds and the structure of gut microbiota (Spearman correlation analysis coefficient). (**A**) The food nutrient content of three bird species. (**B**) The correlation between food nutrient and genus abundance. (f_means family level). **(C)** The relative abundance of *Lactobacillus* and *Psychrobacter* across different waterbirds. Data are means ± SEM. **p < 0.01.

The daily energy intake of the three species of birds was different because of their different feeding habits (Table S1). Considering the food nutrient content, the differences were mainly reflected in: Protein, fat, fibre and carbohydrate (Figure 3A). Through Spearman correlation test, we found that the gut microbiota of birds was closely related to food nutrient composition. *Planococcus* and *Psychrobacter* were positively correlated with fat and protein, while negatively correlated with fibre, ash (minerals) and carbohydrate (Figure 3B, Table S5). Black‐necked cranes possessed a gut microbiota optimized for the digestion of protein and fat, as evidenced by a significant increase in its *Lactobacillus* and *Psychrobacter* (Figure 3C).

**FIGURE 4:**
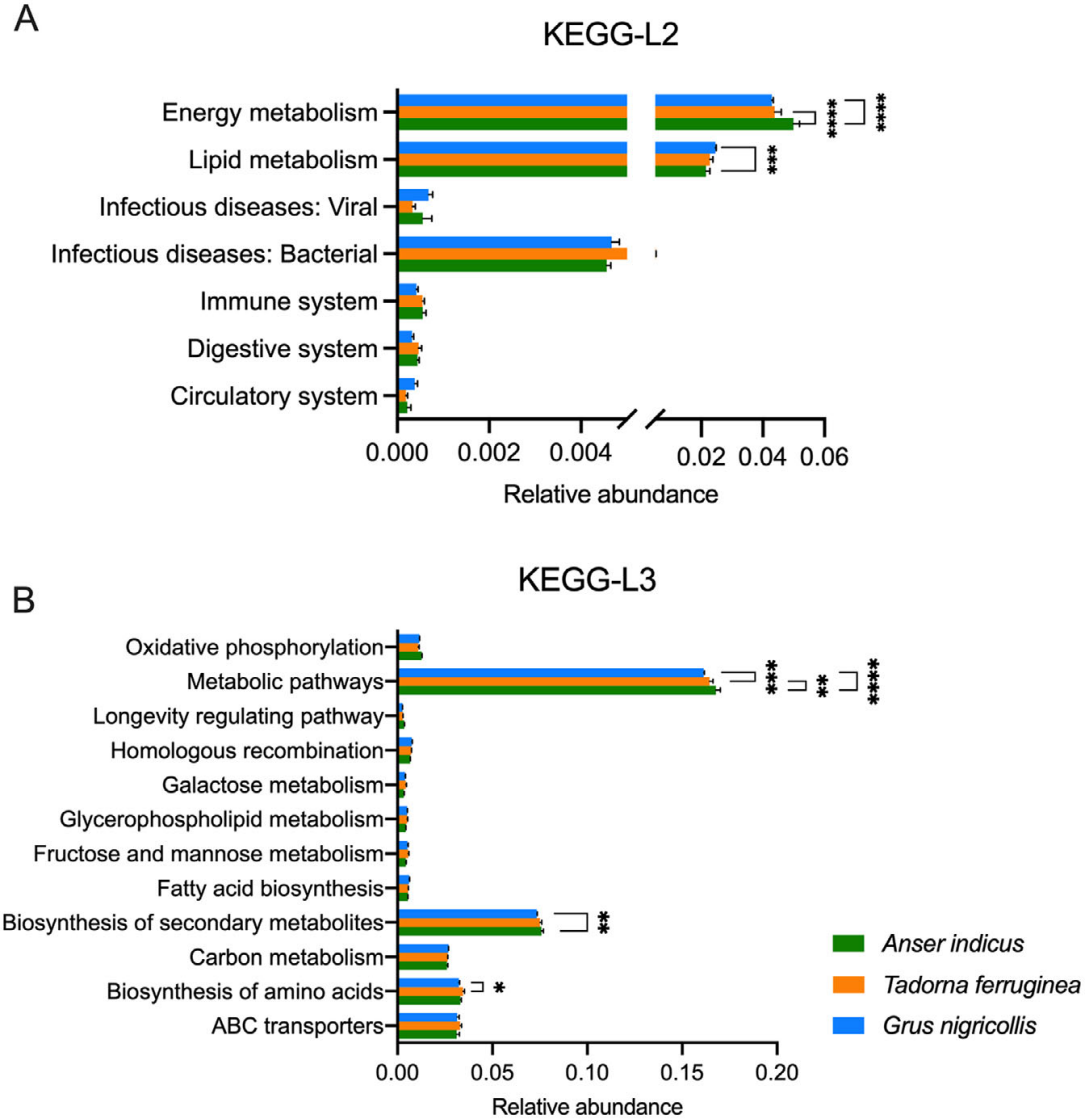
The predicted functional potential of bacterial assemblages associated to each faecal sample was predicted with PICRUSt. (**A**) Difference of prediction function among three species in level 2. (**B**) Difference of prediction function among three species in Level 3. Data are means ± SEM. Two way ANOVA with Tukey's multiple comparisons test, *p < 0.05, **p < 0.01, ***p < 0.001, ****p < 0.0001.

The predicted functional potential of bacterial assemblages associated to each faecal sample was predicted with PICRUSt using Level 2 and 3 of KEGG orthologs. The gut microbiome of samples collected from different species harboured significantly different functional categories. The numbers of bacterial assemblage involved in energy metabolism pathway of the bar‐headed geese was the higher, while the numbers of bacterial assemblage involved in lipid metabolism pathway was the higher in the black‐necked cranes (Figure 4A, Table S6). The numbers of bacterial assemblage involved in biosynthesis of amino acid pathway was the higher in the ruddy shelducks and the bacteria related with biosynthesis of secondary metabolites pathway was the higher in bar‐headed geese (Figure 4B, Table S6). We also observed several common potentially pathogenic genera of bacteria, including *Escherichia‐Shigella*, *Staphylococcus*, *Helicobacter*, *Chryseobacterium*, *Lysinibacillus* and *Carnobacterium*. *Escherichia‐Shigella*, and *Staphylococcus* accounted for a relatively high proportion, especially in ruddy shelducks. *Carnobacterium* was abundant in black‐necked cranes (Figure S2E).

## 4 Discussion

We found that the microbial composition of the three waterbird species was relatively similar, with many shared OTUs. The dominant phyla were Cyanobacteria, Firmicutes and Proteobacteria. Proteobacteria were abundant in all three species, which is consistent with previous findings in wild birds (Bo et al. 2022; Wang et al. 2020). Moreover, when comparing with published data on wild individuals, some differences appear (Zhao et al. 2025). In bar‐headed geese (*Anser indicus*), the relative abundance of Cyanobacteria was higher in zoo individuals. In contrast, ruddy shelducks (*Tadorna ferruginea*) showed a lower proportion of Proteobacteria in captivity than in the wild. This suggests that the complexity of the wild environment may lead to a higher proportion of Proteobacteria compared to captive settings. However, these observations remain limited and would benefit from confirmation through studies with larger sample sizes and better control of confounding factors.

Prior research has identified Proteobacteria as a phylum encompassing a wide array of opportunistic pathogenic genera, such as Campylobacter, Escherichia, Helicobacter, Rickettsia, Salmonella and Vibrio (Keller et al. 2011; Ryu et al. 2014). The normal microbial community within the gut plays a pivotal role in host health maintenance through its interactions with potential pathogens (Eeckhaut et al. 2016). Despite the relatively sanitized feed provided in captive breeding environments, our study still detected the presence of various opportunistic pathogens across the three bird species. It is worth noting that captive black necked cranes exhibit lower levels of these opportunistic pathogenic bacterial genera, which is related to the lack of direct contact with other wild waterbird species in the cage environment. However, this correlation must be interpreted with caution. There are significant differences between captive environments and artificial lake environments in many aspects, including but not limited to feed sources and quality, drinking water cleanliness, population density, hygiene management frequency, potential preventive medication and levels of environmental stressors. The increased prevalence of pathogenic bacteria in the two remaining species of waterbird housed in artificial lakes can be attributed to several factors. First, these artificial lakes allow for free flight of numerous birds, thus increasing the likelihood of pathogen contact. Second, some birds in the artificial lake will migrate between several nearby artificial lakes in the urban area. Finally, human activities and tourist‐related environmental pollution are additional contributors to the introduction and proliferation of opportunistic pathogens within these artificial lakes. Understanding these dynamics is essential for managing the health risks for captive bird populations, particularly in zoo settings where unique challenges to maintaining a stable gut microbiota exist.

The alpha diversity of the gut microbiota in ruddy shelducks and bar‐headed geese was slightly elevated compared to that observed in black‐necked cranes. This may be due to the species itself and dietary differences. The PCoA analysis revealed that each faecal sample had a distinct microbial composition. However, the gut microbiota of ruddy shelducks and bar‐headed geese were more similar to each other and clearly separated from that of the black‐necked cranes. This pattern likely reflects their shared environment and comparable diets. A similar trend has been observed in poultry, where chickens of different breeds raised under the same conditions tend to have similar gut microbiota (Ding et al. 2017). Similarly, in our study, ruddy shelducks and bar‐headed geese, both residing in the same environment and consuming comparable diets, exhibited more similarity in their gut microbiota. By contrast, the black‐necked crane's diet included a significant increasely more in animal food, resulting in a higher abundance of bacteria adapted to such a dietary intake. This observation aligns with previous bird studies, which suggest that food habits primarily influence the community structure and abundance of gut microbiota. However, it is also indisputable that the genetic and taxonomic distance between the black‐necked crane and the other two species plays a role. Host genes are among the crucial factors that impact gut microbial communities (Bo et al. 2022; Bodawatta et al. 2021).

The analysis of the microbial functions associated with three avian species using the PICRUSt method, revealed a connection between their gut microbiota and metabolic pathways involved in energy and lipid metabolism. The differential digestibility of carbohydrates, proteins and cellulose fibre by various microbial taxa suggests that the composition of dietary intake significantly influences shifts in the host's intestinal microbial community. Microbial inhabitants of the Firmicutes and Proteobacteria genera are capable of synthesizing an array of digestive enzymes, enabling the breakdown of diverse cellulose and hemicellulose compounds, and thus facilitating the assimilation of nutritional components from ingested plant material (Morrison et al. 2009). Firmicutes play a pivotal role in the generation of short‐chain fatty acids (SCFAs), with butyrate being a notable example. Butyrate is recognized for its health‐promoting properties, contributing to the host's efficient nutrient utilization and caloric extraction from ingested food. The Firmicutes/Bacteroidetes ratio has been linked to enhanced energy extraction from ingested nutrients in some species, such as humans (Clarke et al. 2012). In our study, we observed that this ratio was notably higher in black‐necked cranes compared to other waterbird species. While direct causal links cannot be established, we speculate that this pattern may relate to species‐specific physiological or ecological factors. First, the diet of black‐necked cranes contains a higher proportion of protein and fat (Ma et al. 2024), which creates an environment favourable to the growth and activity of Firmicutes. Second, as cranes living at high altitudes (2000–4000 m), they rely on Firmicutes to enhance their food digestion and energy extraction. This helps them maintain metabolic balance in hypoxic and cold environments (Liu et al. 2020). Despite their current habitat within low‐altitude zoo, the cranes’ gut microbiota remains influenced by host genetic factors. Our investigation revealed a significantly elevated presence of *Lactobacillus* in black‐necked cranes as opposed to bar‐headed geese and ruddy shelducks. Prior research has characterized *Lactobacillus* as a beneficial intestinal bacterium, conferring anti‐inflammatory effects and pathogen resistance capabilities (Bernardeau et al. 2006). Correlation analysis further demonstrated a positive association between *Planococcus* and *Psychrobacter* abundance and the protein and fat content of their diet, and these two bacteria were significantly high abundance in the digestive tract of black‐necked cranes, which indicated the significant impact of dietary factors on gut microbiota. Although bar‐headed geese and ruddy shelducks exhibit comparable dietary habits and living in the same artificial lake, the ruddy shelducks display increased levels of *Psychrobacter* (Table S4), potentially linked to the consumption of beef within their diet. Therefore, while similarities in living environments and species relationships are important for the composition and structure of gut microbiota, food factors (even a small amount of protein addition) can have an impact on key gut bacteria.

This study has inherent limitations. Avian gut microbiomes are shaped by multifaceted factors including host genetics, environment and diet. Although we selected three waterfowl species co‐housed in the same zoo environment—black‐necked crane (*Grus nigricollis*), bar‐headed goose (*A. indicus*) and ruddy shelduck (*T. ferruginea*)—to minimize zoo‐specific variables, significant phylogenetic divergence exists between these taxa. The crane belongs to the family Gruidae, while the goose and shelduck are members of Anatidae but reside in distinct genera (*Anser* vs. *Tadorna*). Consequently, inherent genetic differences and species‐specific traits (e.g., feeding ecology, behaviour) remain unresolvable confounders that cannot be eliminated by shared captivity. Furthermore, the zoo environment itself—characterized by artificial diets, restricted space, standardized hygiene protocols and reduced habitat complexity—fundamentally differs from natural ecosystems. This limits extrapolation of our findings to wild populations. Future research investigating specific drivers like diet should prioritize closely related Anatidae species or subspecies under controlled captive conditions to isolate target variables more effectively. Nevertheless, captive studies provide valuable insights into host–microbiota interactions under controlled conditions, which can inform conservation and management strategies for captive birds. In addition to the factors mentioned in this article that affect captive animals, it seems that we should also consider the impact of vaccination on the gut microbiota and physiological status of zoo animals in the future.

## 5 Conclusions

In conclusion, this study described the composition, diversity and function of gut microbiome of the *A. indicus*, *T. ferruginea* and *G. nigricollis* in artificial lakes and cages. For waterbirds kept in both captive and semi‐natural conditions, diet is a key factor determining their gut microbiota. *A. indicus* and *T. ferruginea* exhibited similar traits of gut microbiota with the same diet, *G. nigricollis*, whose food primarily consists of fish and meat, possessed a gut microbiota optimized for the digestion of protein and fat. Our study played a bridging role in understanding the differences in the digestive and metabolic abilities between captive animals and wild animals.

## Author Contributions

T.B. and H.L. designed the experiment; J.L., H.Z., Y.Z., and H.L. were responsible for the zoo work; T.B. and H.L. contributed to the analysis of the data. T.B. and H.L. wrote and revised the manuscript. All authors have read and agreed to the published version of the manuscript.

## Consent

The authors have nothing to report.

## Conflicts of Interest

The authors declare no conflicts of interest.

## Supporting information




**Table S1**: Diet composition of three species of studied birds.
**Table S2**: Statistics for the results of data processing from sample sequencing.
**Table S3**: Summary of the numbers of OTU and numbers of sequences in each sample.
**Table S4**: The top ten most abundant core genera of bacteria in the three species of birds.
**Table S5**: Correlation between food composition and genus abundance (rS‐Spearman rank correlation coefficient).
**Table S6**: The difference of predicted functional potential of bacterial assemblages with multiple comparisons.
**Figure S1**: Photos of the living environment of waterbirds; Figure S2: Analysis of differences in gut microbiota among three bird species.

## Data Availability

The raw sequencing data generated in this study have been deposited in the NCBI Sequence Read Archive under the accession numbers PRJNA1102890.
