# Supplementary material for: Comparative Analysis of Gut Microbiota Among Captive Waterbird Species: Effects of Diet and Environmental Factors
Source: Vet Med Sci. 2026 Apr 13;12(3):e70865. doi: 10.1002/vms3.70865 (PMC13075262; doi:10.1002/vms3.70865)
Supplement: Supplementary file 1 — Table S1: Diet composition of three species of studied birds. Table S2: Statistics for the results of data processing from sample sequencing. Table S3: Summary of the numbers of OTU and numbers of sequences in each sample. Table S4: The top ten most abundant core genera of bacteria in the three species of birds. Table S5: Correlation between food composition and genus abundance (rS‐Spearman rank correlation coefficient). Table S6: The difference of predicted functional potential of bacterial assemblages with multiple comparisons. Figure S1: Photos of the living environment of waterbirds; Figure S2: Analysis of differences in gut microbiota among three bird species. [file VMS3-12-e70865-s001.docx]

Comparative Analysis of Gut Microbiota Among Captive Waterbird Species: Effects of Diet and Environmental Factors

Supplementary Materials

Table S1. Diet composition of three species of studied birds.

| Diet (g/day) | Bar-headed goose | Ruddy shelduck | Black-necked crane |
| --- | --- | --- | --- |
| Beef |  | 37.5 | 50 |
| Rape | 120 | 20 | 50 |
| Egg | 50 | 37.5 | 50 |
| Raw fish |  |  | 200 |
| Steamed buns |  |  | 100 |
| Dry corn |  |  | 70 |
| Carrot | 30 | 30 |  |
| Pellet feed* | 120 | 75 |  |

*Pellet feed content：corn (50%), barley (10%), soybean meal (20%), bran(10%), others(10%)

Diet composition of three species of studied birds.

| Composition | Bar-headed goose | Ruddy shelduck | Black-necked crane |
| --- | --- | --- | --- |
| Protein | 23.25% | 29.071% | 36.797% |
| Fat | 6.876% | 8.230% | 9.851% |
| Fiber | 3.804% | 2.361% | 1.828% |
| Carbohydrate | 59.836% | 53.673% | 47.460% |
| Ash | 8.210% | 7.385% | 5.778% |

Table S2. Statistics for the results of data processing from sample sequencing.

| Sample ID | Raw Reads | Clean Reads | Effective Reads | AvgLen(bp) | GC(%) | Q20(%) | Q30(%) | Effective(%) |
| --- | --- | --- | --- | --- | --- | --- | --- | --- |
| ZB1 | 80525 | 80271 | 71005 | 413 | 55.04 | 99.3 | 96.91 | 88.18 |
| ZB2 | 80134 | 79870 | 73173 | 414 | 54.91 | 99.25 | 96.75 | 91.31 |
| ZB3 | 79699 | 79435 | 72660 | 412 | 55.1 | 99.23 | 96.7 | 91.17 |
| ZB4 | 79890 | 79609 | 72370 | 414 | 54.71 | 99.25 | 96.76 | 90.59 |
| ZB5 | 79935 | 79692 | 69249 | 426 | 53.59 | 99.14 | 96.35 | 86.63 |
| ZB6 | 79282 | 79020 | 74687 | 412 | 55.25 | 99.23 | 96.68 | 94.2 |
| ZC1 | 80102 | 79847 | 68724 | 428 | 54.08 | 99.19 | 96.5 | 85.8 |
| ZC2 | 79753 | 79494 | 68317 | 422 | 53.77 | 99.21 | 96.6 | 85.66 |
| ZC3 | 79992 | 79754 | 69447 | 422 | 53.99 | 99.16 | 96.43 | 86.82 |
| ZC4 | 80204 | 79962 | 69261 | 421 | 54.39 | 99.22 | 96.63 | 86.36 |
| ZC5 | 80083 | 79818 | 74602 | 412 | 54.45 | 99.22 | 96.67 | 93.16 |
| ZC6 | 80094 | 79818 | 71541 | 414 | 53.37 | 99.24 | 96.74 | 89.32 |
| ZH1 | 73080 | 72841 | 70703 | 426 | 52.05 | 99.17 | 96.39 | 96.75 |
| ZH10 | 79813 | 79537 | 64290 | 418 | 54.28 | 99.18 | 96.52 | 80.55 |
| ZH11 | 79649 | 79376 | 73578 | 422 | 52.61 | 99.13 | 96.31 | 92.38 |
| ZH12 | 80066 | 79769 | 72660 | 417 | 54.9 | 99.24 | 96.7 | 90.75 |
| ZH13 | 80051 | 79773 | 72412 | 422 | 53.71 | 99.2 | 96.56 | 90.46 |
| ZH2 | 80071 | 79804 | 76551 | 427 | 52.08 | 99.21 | 96.55 | 95.6 |
| ZH3 | 79912 | 79622 | 67077 | 424 | 52.87 | 99.17 | 96.43 | 83.94 |
| ZH4 | 80004 | 79753 | 76981 | 422 | 53.47 | 99.23 | 96.59 | 96.22 |
| ZH5 | 79989 | 79750 | 66868 | 429 | 53.28 | 99.21 | 96.5 | 83.6 |
| ZH6 | 79881 | 79660 | 77731 | 423 | 53.09 | 99.2 | 96.49 | 97.31 |
| ZH7 | 80303 | 80043 | 78246 | 421 | 53.92 | 99.18 | 96.48 | 97.44 |
| ZH8 | 79821 | 79573 | 72366 | 419 | 54.27 | 99.17 | 96.46 | 90.66 |
| ZH9 | 79662 | 79412 | 69928 | 417 | 53.54 | 99.22 | 96.64 | 87.78 |

Table S3. Summary of the numbers of OTU and numbers of sequences in each sample.

| Sample ID | OTU_Number | Seqs_Number | Species |
| --- | --- | --- | --- |
| ZB1 | 940 | 45221 | Bar-headed goose |
| ZB2 | 886 | 51435 | Bar-headed goose |
| ZB3 | 950 | 53403 | Bar-headed goose |
| ZB4 | 925 | 52887 | Bar-headed goose |
| ZB5 | 784 | 62326 | Bar-headed goose |
| ZB6 | 922 | 58472 | Bar-headed goose |
| ZC1 | 759 | 60731 | Ruddy shelduck |
| ZC2 | 1046 | 57580 | Ruddy shelduck |
| ZC3 | 881 | 58118 | Ruddy shelduck |
| ZC4 | 999 | 50679 | Ruddy shelduck |
| ZC5 | 782 | 56516 | Ruddy shelduck |
| ZC6 | 838 | 53219 | Ruddy shelduck |
| ZH1 | 872 | 66438 | Black-necked crane |
| ZH10 | 891 | 48453 | Black-necked crane |
| ZH11 | 893 | 63842 | Black-necked crane |
| ZH12 | 1212 | 54713 | Black-necked crane |
| ZH13 | 1141 | 56286 | Black-necked crane |
| ZH2 | 711 | 72261 | Black-necked crane |
| ZH3 | 654 | 59962 | Black-necked crane |
| ZH4 | 1132 | 65550 | Black-necked crane |
| ZH5 | 599 | 60825 | Black-necked crane |
| ZH6 | 914 | 73307 | Black-necked crane |
| ZH7 | 1126 | 68795 | Black-necked crane |
| ZH8 | 897 | 51043 | Black-necked crane |
| ZH9 | 936 | 54782 | Black-necked crane |
| Total | 1812 | 1456844 |  |

Table S4. The top ten most abundant core genera of bacteria in the three species of birds.

| Genus | Black-necked crane | Ruddy shelduck | Bar-headed goose |
| --- | --- | --- | --- |
| uncultured_bacterium_o_Chloroplast | 0.040046116 | 0.111200173 | 0.298810171 |
| Psychrobacter | 0.255544378 | 0.00611264 | 0.000401552 |
| Lactobacillus | 0.168723164 | 0.025427276 | 0.016849733 |
| Acinetobacter | 0.026699922 | 0.075967142 | 0.099967876 |
| Enterobacter | 0.013671465 | 0.059383749 | 0.01742735 |
| Lactococcus | 0.001358858 | 0.084511182 | 0.001473386 |
| uncultured_bacterium_f_Enterobacteriaceae | 0.00941279 | 0.056700006 | 0.016426559 |
| Escherichia-Shigella | 0.02526948 | 0.045703785 | 0.006922136 |
| Enterococcus | 0.011073058 | 0.06018234 | 0.002217802 |
| Sphingomonas | 0.0229549 | 0.00847279 | 0.040667935 |

Table S5. Correlation between food composition and genus abundance (Spearman rank correlation coefficient).

| Genus | Component | cor | pvalue | adj.p | significance |
| --- | --- | --- | --- | --- | --- |
| Psychrobacter | Fiber | -0.675381634 | 0.000211879 | 0.031146186 | * |
| Psychrobacter | Protein | 0.847415046 | 9.07E-08 | 1.41E-05 | *** |
| Planococcus | Protein | 0.811596906 | 8.50E-07 | 0.000128412 | *** |
| Psychrobacter | Fat | 0.847415046 | 9.07E-08 | 1.41E-05 | *** |
| Planococcus | Fat | 0.811596906 | 8.50E-07 | 0.000128412 | *** |
| Psychrobacter | Carbohydrate | -0.847415046 | 9.07E-08 | 1.41E-05 | *** |
| Planococcus | Carbohydrate | -0.811596906 | 8.50E-07 | 0.000128412 | *** |
| Psychrobacter | Ash | -0.847415046 | 9.07E-08 | 1.41E-05 | *** |
| Planococcus | Ash | -0.811596906 | 8.50E-07 | 0.000128412 | *** |

Table S6. The difference of predicted functional potential of bacterial assemblages with multiple comparisons.

KEGG L2

| Tukey's multiple comparisons test | Predicted (LS) mean diff. | 95.00% CI of diff. | Summary | Adjusted P Value |
| --- | --- | --- | --- | --- |
|  |  |  |  |  |
| Circulatory system |  |  |  |  |
| *Anser indicus* vs. *Tadorna ferruginea* | 3.168e-005 | -0.002144 to 0.002208 | ns | 0.9993 |
| *Anser indicus* vs. *Grus nigricollis* | -0.0001620 | -0.002022 to 0.001698 | ns | 0.9769 |
| *Tadorna ferruginea* vs. *Grus nigricollis* | -0.0001936 | -0.002054 to 0.001667 | ns | 0.9671 |
|  |  |  |  |  |
| Digestive system |  |  |  |  |
| *Anser indicus* vs. *Tadorna ferruginea* | -2.409e-005 | -0.002200 to 0.002152 | ns | 0.9996 |
| *Anser indicus* vs. *Grus nigricollis* | 0.0001181 | -0.001742 to 0.001978 | ns | 0.9876 |
| *Tadorna ferruginea* vs. *Grus nigricollis* | 0.0001421 | -0.001718 to 0.002002 | ns | 0.9821 |
|  |  |  |  |  |
| Immune system |  |  |  |  |
| *Anser indicus* vs. *Tadorna ferruginea* | 4.984e-006 | -0.002171 to 0.002181 | ns | >0.9999 |
| *Anser indicus* vs. *Grus nigricollis* | 0.0001398 | -0.001720 to 0.002000 | ns | 0.9827 |
| *Tadorna ferruginea* vs. *Grus nigricollis* | 0.0001349 | -0.001725 to 0.001995 | ns | 0.9839 |
|  |  |  |  |  |
| Infectious diseases: Bacterial |  |  |  |  |
| *Anser indicus* vs. *Tadorna ferruginea* | -0.0005424 | -0.002719 to 0.001634 | ns | 0.8257 |
| *Anser indicus* vs. *Grus nigricollis* | -0.0001103 | -0.001971 to 0.001750 | ns | 0.9892 |
| *Tadorna ferruginea* vs. *Grus nigricollis* | 0.0004321 | -0.001428 to 0.002292 | ns | 0.8468 |
|  |  |  |  |  |
| Infectious diseases: Viral |  |  |  |  |
| *Anser indicus* vs. *Tadorna ferruginea* | 0.0002199 | -0.001956 to 0.002396 | ns | 0.9690 |
| *Anser indicus* vs. *Grus nigricollis* | -0.0001266 | -0.001987 to 0.001734 | ns | 0.9858 |
| *Tadorna ferruginea* vs. *Grus nigricollis* | -0.0003465 | -0.002207 to 0.001514 | ns | 0.8985 |
|  |  |  |  |  |
| Lipid metabolism |  |  |  |  |
| *Anser indicus* vs. *Tadorna ferruginea* | -0.001255 | -0.003431 to 0.0009208 | ns | 0.3617 |
| *Anser indicus* vs. *Grus nigricollis* | -0.003025 | -0.004885 to -0.001164 | *** | 0.0005 |
| *Tadorna ferruginea* vs. *Grus nigricollis* | -0.001769 | -0.003630 to 9.097e-005 | ns | 0.0660 |
|  |  |  |  |  |
| Energy metabolism |  |  |  |  |
| *Anser indicus* vs. *Tadorna ferruginea* | 0.006061 | 0.003884 to 0.008237 | **** | <0.0001 |
| *Anser indicus* vs. *Grus nigricollis* | 0.007035 | 0.005175 to 0.008895 | **** | <0.0001 |
| *Tadorna ferruginea* vs. *Grus nigricollis* | 0.0009743 | -0.0008860 to 0.002835 | ns | 0.4318 |

KEGG L3

| Tukey's multiple comparisons test | Predicted (LS) mean diff. | 95.00% CI of diff. | Summary | Adjusted P Value |
| --- | --- | --- | --- | --- |
|  |  |  |  |  |
| ABC transporters |  |  |  |  |
| *Anser indicus* vs. *Tadorna ferruginea* | -0.001881 | -0.004148 to 0.0003859 | ns | 0.1254 |
| *Anser indicus* vs. *Grus nigricollis* | -0.0003488 | -0.002287 to 0.001589 | ns | 0.9056 |
| *Tadorna ferruginea* vs. *Grus nigricollis* | 0.001532 | -0.0004057 to 0.003471 | ns | 0.1514 |
|  |  |  |  |  |
| Biosynthesis of amino acids |  |  |  |  |
| *Anser indicus* vs. *Tadorna ferruginea* | -0.001423 | -0.003690 to 0.0008440 | ns | 0.3024 |
| *Anser indicus* vs. *Grus nigricollis* | 0.0007509 | -0.001187 to 0.002689 | ns | 0.6324 |
| *Tadorna ferruginea* vs. *Grus nigricollis* | 0.002174 | 0.0002360 to 0.004112 | * | 0.0235 |
|  |  |  |  |  |
| Carbon metabolism |  |  |  |  |
| *Anser indicus* vs. *Tadorna ferruginea* | -0.0002855 | -0.002553 to 0.001982 | ns | 0.9526 |
| *Anser indicus* vs. *Grus nigricollis* | -0.0008263 | -0.002764 to 0.001112 | ns | 0.5745 |
| *Tadorna ferruginea* vs. *Grus nigricollis* | -0.0005408 | -0.002479 to 0.001397 | ns | 0.7882 |
|  |  |  |  |  |
| Biosynthesis of secondary metabolites |  |  |  |  |
| *Anser indicus* vs. *Tadorna ferruginea* | 0.0008250 | -0.001442 to 0.003092 | ns | 0.6674 |
| *Anser indicus* vs. *Grus nigricollis* | 0.002544 | 0.0006063 to 0.004483 | ** | 0.0062 |
| *Tadorna ferruginea* vs. *Grus nigricollis* | 0.001719 | -0.0002187 to 0.003658 | ns | 0.0936 |
|  |  |  |  |  |
| Fatty acid biosynthesis |  |  |  |  |
| *Anser indicus* vs. *Tadorna ferruginea* | -0.0002018 | -0.002469 to 0.002065 | ns | 0.9760 |
| *Anser indicus* vs. *Grus nigricollis* | -0.0007580 | -0.002696 to 0.001180 | ns | 0.6270 |
| *Tadorna ferruginea* vs. *Grus nigricollis* | -0.0005562 | -0.002494 to 0.001382 | ns | 0.7775 |
|  |  |  |  |  |
| Fructose and mannose metabolism |  |  |  |  |
| *Anser indicus* vs. *Tadorna ferruginea* | -0.001280 | -0.003547 to 0.0009873 | ns | 0.3795 |
| *Anser indicus* vs. *Grus nigricollis* | -0.0009702 | -0.002908 to 0.0009679 | ns | 0.4662 |
| *Tadorna ferruginea* vs. *Grus nigricollis* | 0.0003097 | -0.001628 to 0.002248 | ns | 0.9248 |
|  |  |  |  |  |
| Glycerophospholipid metabolism |  |  |  |  |
| *Anser indicus* vs. *Tadorna ferruginea* | -0.0008334 | -0.003101 to 0.001434 | ns | 0.6620 |
| *Anser indicus* vs. *Grus nigricollis* | -0.0008091 | -0.002747 to 0.001129 | ns | 0.5876 |
| *Tadorna ferruginea* vs. *Grus nigricollis* | 2.424e-005 | -0.001914 to 0.001962 | ns | 0.9995 |
|  |  |  |  |  |
| Galactose metabolism |  |  |  |  |
| *Anser indicus* vs. *Tadorna ferruginea* | -0.001009 | -0.003276 to 0.001258 | ns | 0.5468 |
| *Anser indicus* vs. *Grus nigricollis* | -0.0006195 | -0.002558 to 0.001319 | ns | 0.7319 |
| *Tadorna ferruginea* vs. *Grus nigricollis* | 0.0003894 | -0.001549 to 0.002327 | ns | 0.8838 |
|  |  |  |  |  |
| Homologous recombination |  |  |  |  |
| *Anser indicus* vs. *Tadorna ferruginea* | -0.0006383 | -0.002905 to 0.001629 | ns | 0.7848 |
| *Anser indicus* vs. *Grus nigricollis* | -0.0009978 | -0.002936 to 0.0009403 | ns | 0.4462 |
| *Tadorna ferruginea* vs. *Grus nigricollis* | -0.0003596 | -0.002298 to 0.001579 | ns | 0.9000 |
|  |  |  |  |  |
| Longevity regulating pathway |  |  |  |  |
| *Anser indicus* vs. *Tadorna ferruginea* | 0.0007885 | -0.001479 to 0.003056 | ns | 0.6911 |
| *Anser indicus* vs. *Grus nigricollis* | 0.0009090 | -0.001029 to 0.002847 | ns | 0.5115 |
| *Tadorna ferruginea* vs. *Grus nigricollis* | 0.0001205 | -0.001818 to 0.002059 | ns | 0.9882 |
|  |  |  |  |  |
| Metabolic pathways |  |  |  |  |
| *Anser indicus* vs. *Tadorna ferruginea* | 0.003446 | 0.001179 to 0.005713 | ** | 0.0012 |
| *Anser indicus* vs. *Grus nigricollis* | 0.006557 | 0.004619 to 0.008495 | **** | <0.0001 |
| *Tadorna ferruginea* vs. *Grus nigricollis* | 0.003111 | 0.001173 to 0.005049 | *** | 0.0006 |
|  |  |  |  |  |
| Oxidative phosphorylation |  |  |  |  |
| *Anser indicus* vs. *Tadorna ferruginea* | 0.001884 | -0.0003836 to 0.004151 | ns | 0.1247 |
| *Anser indicus* vs. *Grus nigricollis* | 0.001258 | -0.0006806 to 0.003196 | ns | 0.2788 |
| *Tadorna ferruginea* vs. *Grus nigricollis* | -0.0006261 | -0.002564 to 0.001312 | ns | 0.7270 |

A B

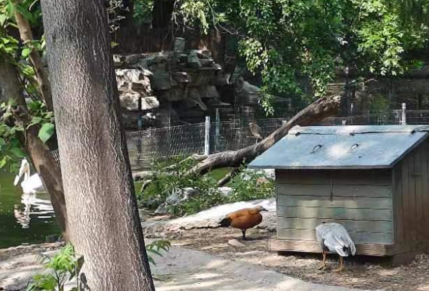

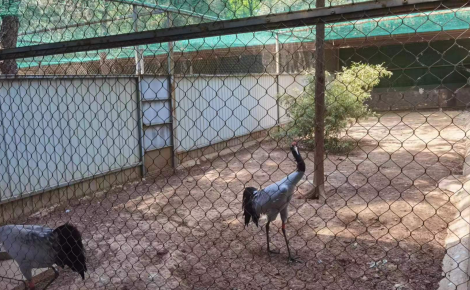


Figure S1 Photos of the living environment of waterbirds.

(A) Living environment of bar-headed goose and ruddy shelduck. (B) Living environment of black-necked crane.


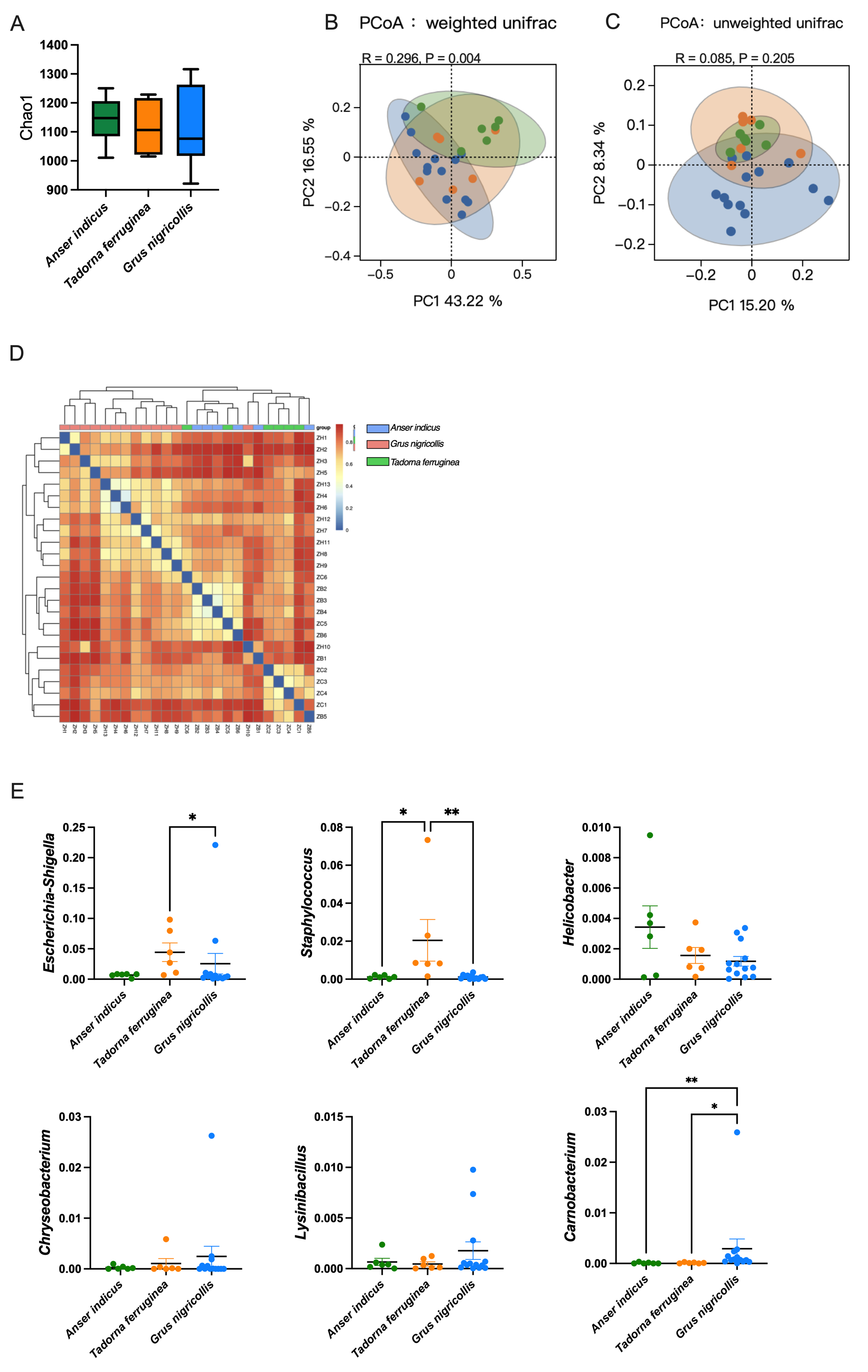


Figure S2 Analysis of differences in gut microbiota among three bird species.

(A) Chao 1 index of 3 bird species. (B,C) The PCoA plots of weighted unifrac and unweighted unifrac distance. (D) Heatmap analysis of Bray-Curtis distance matrix between samples. (E) The relative abundance of 6 microbiota genera across three different species of waterbirds.
